# Surfactant disaturated-phosphatidylcholine kinetics in acute respiratory distress syndrome by stable isotopes and a two compartment model

**DOI:** 10.1186/1465-9921-8-13

**Published:** 2007-02-21

**Authors:** Paola E Cogo, Gianna Maria Toffolo, Carlo Ori, Andrea Vianello, Marco Chierici, Antonina Gucciardi, Claudio Cobelli, Aldo Baritussio, Virgilio P Carnielli

**Affiliations:** 1 Department of Pediatrics, University of Padova, Padova, Italy; 2 Department of Information Engineering, University of Padova, Italy; 3 Department of Pharmacology, Anaesthesia and Critical Care, University of Padova, Padova, Italy; 4 Respiratory Unit, General Medical Hospital, Padova, Italy; 5 Department of Medical and Surgical Sciences, University of Padova, Padova, Italy; 6 Neonatal Division, Salesi Children's Hospital, Ancona, Italy; 7 Nutrition Unit, Institute of Child Health and Great Ormond Street Hospital, London, UK

## Abstract

**Background:**

In patients with acute respiratory distress syndrome (ARDS), it is well known that only part of the lungs is aerated and surfactant function is impaired, but the extent of lung damage and changes in surfactant turnover remain unclear. The objective of the study was to evaluate surfactant disaturated-phosphatidylcholine turnover in patients with ARDS using stable isotopes.

**Methods:**

We studied 12 patients with ARDS and 7 subjects with normal lungs. After the tracheal instillation of a trace dose of ^13^C-dipalmitoyl-phosphatidylcholine, we measured the ^13^C enrichment over time of palmitate residues of disaturated-phosphatidylcholine isolated from tracheal aspirates. Data were interpreted using a model with two compartments, alveoli and lung tissue, and kinetic parameters were derived assuming that, in controls, alveolar macrophages may degrade between 5 and 50% of disaturated-phosphatidylcholine, the rest being lost from tissue. In ARDS we assumed that 5–100% of disaturated-phosphatidylcholine is degraded in the alveolar space, due to release of hydrolytic enzymes. Some of the kinetic parameters were uniquely determined, while others were identified as lower and upper bounds.

**Results:**

In ARDS, the alveolar pool of disaturated-phosphatidylcholine was significantly lower than in controls (0.16 ± 0.04 vs. 1.31 ± 0.40 mg/kg, p < 0.05). Fluxes between tissue and alveoli and *de novo *synthesis of disaturated-phosphatidylcholine were also significantly lower, while mean resident time in lung tissue was significantly higher in ARDS than in controls. Recycling was 16.2 ± 3.5 in ARDS and 31.9 ± 7.3 in controls (p = 0.08).

**Conclusion:**

In ARDS the alveolar pool of surfactant is reduced and disaturated-phosphatidylcholine turnover is altered.

## Background

ARDS is a syndrome of reduced gas exchange due to a diffuse injury to the alveolar capillary barrier and is characterized by filling of the alveoli with proteinaceous fluid, infiltration by inflammatory cells and consolidation [1]. It may develop after a direct insult to the lung parenchyma or it may result from inflammatory processes carried into the lungs via the pulmonary vasculature. In the early exudative phase of ARDS the massive, self-perpetuating inflammatory process is characterized by an increased endothelial and epithelial permeability with leakage of plasma components.

Constriction and microembolism of the pulmonary vessels are also present, leading to ventilation perfusion mismatch. Moreover an increase in the alveolar surface tension causes alveolar instability, atelectasis and ventilatory inhomogenieties. In severe ARDS, just a small fraction of parenchyma remains aerated, and the damage can be so widespread that normal parenchyma, as judged by computed tomography, may shrink to 200–500 g [2,3].

One of the hallmarks of ARDS is reduced lung compliance and loss of stability of terminal airways at low volumes, suggesting surfactant dysfunction or deficiency. Samples of bronchoalveolar lavage fluid from patients with ARDS have low concentrations of disaturated-phosphatidylcholine, phosphatidylglycerol and surfactant-specific proteins and fail to reduce surface tension both *in vitro *and *in vivo *[4,5]. Surfactant organization in the alveoli is also altered, since large aggregates, the active fraction of surfactant, decrease in patients with ARDS [6]. To our knowledge, the alveolar pool of surfactant has never been rigorously estimated in patients with ARDS, nor is it known if surfactant turnover is altered in this condition.

Data on surfactant metabolism in ARDS are available from animal studies which showed a faster turnover rate and a decreased alveolar pool of disaturated-phosphatidylcholine, while the tissue pool was increased in some studies and unchanged in others [7-9]. However these experiments cannot be repeated in humans and may not necessarily mimic human disease.

In this paper we studied the turnover of surfactant disaturated-phosphatidylcholine in patients with ARDS and in control subjects. To this end we instilled a trace dose of ^13^C-dipalmitoyl-phosphatidylcholine into the trachea and then followed over time the ^13^C enrichments in disaturated-phosphatidylcholine-palmitate isolated from serial tracheal aspirates.

Available evidence indicates that surfactant dipalmitoyl-phosphatidylcholine is recycled several times before being degraded by alveolar macrophages or within lung parenchyma [7]. There is uncertainty, however, about the contribution of alveolar macrophages to surfactant catabolism, since animal experiments indicate that alveolar macrophages could degrade between 5 and 50% of surfactant disaturated-phosphatidylcholine [10,11]. In patients with ARDS, the fraction of disaturated-phosphatidylcholine degraded in the alveolar space could be even greater than this, due to the presence of inflammatory cells, bacteria and free hydrolytic enzymes [12,13]. On the basis of these considerations we assumed that alveolar macrophages may degrade 5–50% of saturated phosphatidylcholine in controls and 5–100% in patients with ARDS.

## Methods

### Patients

We studied 12 adult patients with ARDS, defined according to Bernard [14], and 7 subjects with normal lungs on mechanical ventilation or breathing spontaneously through a tracheostomy tube due to neuromuscular diseases. Patients were admitted to the Intensive Care or Respiratory Units of the University of Padova, Italy. The study was approved by the Ethics Committee, and written, informed consent was obtained. After intubation with a cuffed tube, all patients received into the trachea 20 ml of normal saline containing 7.5 mg of ^13^C-dipalmitoyl-phosphatidylcholine and 40 mg of surfactant extract (Curosurf^®^, Chiesi, Parma, Italy) as spreading agent. Both palmitates were uniformly labeled with carbon 13 ([U-^13^C-PA]-DPPC, Martek-Biosciences, Columbia, MD). The suspension was instilled close to the carina with a 4.5 mm bronchoscope (Olympus BF-40 OD 6.0 mm Olympus-Europe, Italy). Patients with ARDS were studied within 72 h from the onset of the acute respiratory failure and ventilator parameters were adjusted to maintain an oxygen saturation > 85% and pH > 7.25. Ventilator and gas exchange parameters were recorded at time 0 and subsequently every 6 h in ARDS patients and at least once in controls.

### Study design

Tracheal aspirates, collected by suction below the tip of the endotracheal tube after instilling 5 ml of normal saline, were obtained at baseline, every 6 h until 72 h and then every 12 h for 7 days or until extubation. Aspirates were filtered on gauze, centrifuged at 150-g for 10 minutes and supernatants were stored at -20°C.

### Analytical methods

Lipids from tracheal aspirates and from the administered tracer were extracted according to Bligh and Dyer after addition of the internal standard heptadecanoylphosphatidylcholine [15]. One third of the extract was oxidized with osmium tetroxide. Disaturated-phosphatidilcholine was isolated from the lipid extract by thin layer chromatography [16], the fatty acids were derivatized as pentafluorobenzyl derivatives [17], extracted with hexane and stored at -20°C. Tracheal aspirates with visible blood were discarded. The enrichments of ^13^C -disaturated-phosphatidylcholine-palmitate were measured by gas chromatography-mass spectrometry (GC-MS, Voyager, Thermoquest, Rodano, Milano, Italy), as previously described [18].

### Data analysis

**Figure 1:**
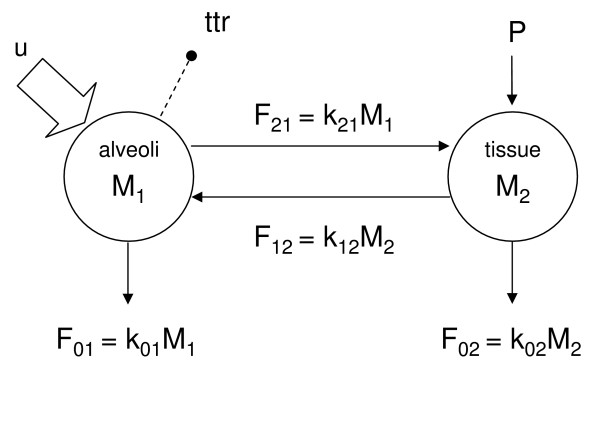
**A two compartment model**. Two compartment model for the analysis of disaturated-phosphatidylcholine-palmitate kinetics. Compartment 1 is the alveolar space, compartment 2 is lung tissue. M_1 _and M_2 _are tracee disaturated-phosphatidylcholine-palmitate masses, P is disaturated-phosphatidylcholine-palmitate *de novo *synthesis, F_21 _and F_12 _are inter-conversion fluxes, F_01 _and F_02 _are irreversible loss fluxes, k_21 _and k_12 _are interconversion rate parameters, k_01 _and k_02 _are irreversible loss rate parameters, u is the tracer disaturated-phosphatidylcholine-palmitate input in compartment 1 and the dashed line with a bullet indicates the tracer to tracee ratio (ttr) measurement. It is assumed that loss from the alveolar space is 5–50% in controls and 5–100% in ARDS.

Data were analyzed with the two compartment model shown in figure 1 under the following assumptions: a) surfactant is distributed between two compartments (alveoli and lung parenchyma); b) disaturated-phosphatidylcholine is synthesized by lung parenchyma, secreted in the alveoli and recycled before being degraded by alveolar macrophages or lung tissue; c) the system is at steady state and is not perturbed by the administration of tracer. These assumptions have been validated in adult and newborn animals by several authors, and have been used in numerous studies on surfactant turnover in experimental animals [7,19-21].

Tracer model equations are:

m˙1
 MathType@MTEF@5@5@+=feaafiart1ev1aaatCvAUfKttLearuWrP9MDH5MBPbIqV92AaeXatLxBI9gBaebbnrfifHhDYfgasaacH8akY=wiFfYdH8Gipec8Eeeu0xXdbba9frFj0=OqFfea0dXdd9vqai=hGuQ8kuc9pgc9s8qqaq=dirpe0xb9q8qiLsFr0=vr0=vr0dc8meaabaqaciaacaGaaeqabaqabeGadaaakeaacuqGTbqBgaGaamaaBaaaleaacqaIXaqmaeqaaaaa@2F32@ (t) = -(k_01 _+ k_21_)m_1 _(t) + k_12_m_2 _(t) + u(t)

m˙1
 MathType@MTEF@5@5@+=feaafiart1ev1aaatCvAUfKttLearuWrP9MDH5MBPbIqV92AaeXatLxBI9gBaebbnrfifHhDYfgasaacH8akY=wiFfYdH8Gipec8Eeeu0xXdbba9frFj0=OqFfea0dXdd9vqai=hGuQ8kuc9pgc9s8qqaq=dirpe0xb9q8qiLsFr0=vr0=vr0dc8meaabaqaciaacaGaaeqabaqabeGadaaakeaacuqGTbqBgaGaamaaBaaaleaacqaIXaqmaeqaaaaa@2F32@ (t) = k_21_m_1 _(t) - (k_01 _+ k_12_)m_2 _(t)     (1)

where m_1 _and m_2 _are the amount (in mg) of disaturated-phosphatidylcholine-palmitate tracer in compartment 1 and 2 respectively, m˙1
 MathType@MTEF@5@5@+=feaafiart1ev1aaatCvAUfKttLearuWrP9MDH5MBPbIqV92AaeXatLxBI9gBaebbnrfifHhDYfgasaacH8akY=wiFfYdH8Gipec8Eeeu0xXdbba9frFj0=OqFfea0dXdd9vqai=hGuQ8kuc9pgc9s8qqaq=dirpe0xb9q8qiLsFr0=vr0=vr0dc8meaabaqaciaacaGaaeqabaqabeGadaaakeaacuqGTbqBgaGaamaaBaaaleaacqaIXaqmaeqaaaaa@2F32@ and m˙2
 MathType@MTEF@5@5@+=feaafiart1ev1aaatCvAUfKttLearuWrP9MDH5MBPbIqV92AaeXatLxBI9gBaebbnrfifHhDYfgasaacH8akY=wiFfYdH8Gipec8Eeeu0xXdbba9frFj0=OqFfea0dXdd9vqai=hGuQ8kuc9pgc9s8qqaq=dirpe0xb9q8qiLsFr0=vr0=vr0dc8meaabaqaciaacaGaaeqabaqabeGadaaakeaacuqGTbqBgaGaamaaBaaaleaacqaIYaGmaeqaaaaa@2F34@ (mg/h) represent their rate of change, k_21 _and k_12 _(h^-1^) are inter-conversion rate parameters, k_01 _and k_02 _(h^-1^) are irreversible losses, and u is the labeled disaturated-phosphatidylcholine-palmitate injection into the accessible compartment.

Tracee steady state equations are:

0 = -(K_01 _+ K_21_)M_1 _+ K_12_M_2 _= -F_01 _- F_21 _+ F_12_

0 = K_21_M_1 _- (K_01 _+ K_12_)M_2 _+ P = F_21 _- F_01 _- F_12 _+ P     (2)

where M_1 _and M_2 _(mg) are the steady state tracee disaturated-phosphatidylcholine-palmitate masses in the two compartments, P (mg/h) is disaturated-phosphatidylcholine-palmitate *de novo *synthesis, F_21 _= k_21_M_1_, F_12 _= k_12_M_2, _F_01 _= k_01_M_1_, F_02 _= k_02_M_2 _(mg/h) are inter-conversion and irreversible loss fluxes.

Measured tracer to tracee ratio at time t is the ratio between tracer and tracee masses in the accessible compartment:

ttr1(t)=m1(t)M1     (3)
 MathType@MTEF@5@5@+=feaafiart1ev1aaatCvAUfKttLearuWrP9MDH5MBPbIqV92AaeXatLxBI9gBaebbnrfifHhDYfgasaacH8akY=wiFfYdH8Gipec8Eeeu0xXdbba9frFj0=OqFfea0dXdd9vqai=hGuQ8kuc9pgc9s8qqaq=dirpe0xb9q8qiLsFr0=vr0=vr0dc8meaabaqaciaacaGaaeqabaqabeGadaaakeaacqqG0baDcqqG0baDcqqGYbGCdaWgaaWcbaGaeGymaedabeaakiabcIcaOiabbsha0jabcMcaPiabg2da9maalaaabaGaeeyBa02aaSbaaSqaaiabigdaXaqabaGccqGGOaakcqqG0baDcqGGPaqkaeaacqqGnbqtdaWgaaWcbaGaeGymaedabeaaaaGccaWLjaGaaCzcamaabmaabaGaeG4mamdacaGLOaGaayzkaaaaaa@4202@

The tracer model (equations 1 and 3) is not identifiable, since it is not possible to quantify from the input-output tracer experiment in the alveolar compartment unique values for the unknown parameters of the tracer model, namely M_1_, k_01_, k_02_, k_12_, k_21 _[22]. Only the mass in the alveolar compartment M_1 _can be uniquely identified, together with some combinations of the original parameters, namely k_01_+ k_21_, k_02 _+ k_21 _and k_21 _k_12_. To resolve model nonidentifiability, assumptions on the relative role of the two degradation pathways need to be incorporated into the model. Based on the results of studies in which rabbits or mice received non-degradable analogues of disaturated-phosphatidylcholine into the trachea [10,11], we assumed that, in normal subjects, alveolar macrophages may degrade between 5 and 50% of surfactant disaturated-phosphatidylcholine, the remaining being degraded by lung parenchyma (i.e. F_01 _varies between 5 and 50% of F_01_+F_02_). In ARDS, we assumed that the degradation of disaturated-phosphatidylcoline in the airways could vary between 5 and 100% due to the degradative activity of inflammatory cells, bacteria or enzymes released in the alveolar spaces (i.e. F_01 _varies between 5 and 100% of F_01_+F_02_). Using this information, upper and lower bounds for parameters k_12_, k_21_, k_01_and k_02 _were estimated from tracer to tracee data in each individual [23]. Using these values in equation 2, upper and lower bounds were derived for P, M_2 _and tracee fluxes F_21 _and F_02_, while flux F_12 _was uniquely solved [22]. Additional kinetic parameters were used to characterize the system, namely the total mass in the system (M_tot _= M_1 _+ M_2_), the mean residence time of molecules entering the system from alveoli or lung tissue (MRT_1_, MRT_2_), defined as the sum of the elements in column 1 and 2 of the mean residence time matrix Θ:

Θ=[−(k01+k21)k12k21−(k02+k12)]−1=1k21k02+k01k02+k01k12[k02+k12k12k21k01+k21]     (4)
 MathType@MTEF@5@5@+=feaafiart1ev1aaatCvAUfKttLearuWrP9MDH5MBPbIqV92AaeXatLxBI9gBaebbnrfifHhDYfgasaacH8akY=wiFfYdH8Gipec8Eeeu0xXdbba9frFj0=OqFfea0dXdd9vqai=hGuQ8kuc9pgc9s8qqaq=dirpe0xb9q8qiLsFr0=vr0=vr0dc8meaabaqaciaacaGaaeqabaqabeGadaaakeaacqqHyoqucqGH9aqpdaWadaqaauaabeqaciaaaeaacqGHsislcqGGOaakcqqGRbWAdaWgaaWcbaGaeGimaaJaeGymaedabeaakiabgUcaRiabbUgaRnaaBaaaleaacqaIYaGmcqaIXaqmaeqaaOGaeiykaKcabaGaee4AaS2aaSbaaSqaaiabigdaXiabikdaYaqabaaakeaacqqGRbWAdaWgaaWcbaGaeGOmaiJaeGymaedabeaaaOqaaiabgkHiTiabcIcaOiabbUgaRnaaBaaaleaacqaIWaamcqaIYaGmaeqaaOGaey4kaSIaee4AaS2aaSbaaSqaaiabigdaXiabikdaYaqabaGccqGGPaqkaaaacaGLBbGaayzxaaWaaWbaaSqabeaacqGHsislcqaIXaqmaaGccqGH9aqpdaWcaaqaaiabigdaXaqaaiabbUgaRnaaBaaaleaacqaIYaGmcqaIXaqmaeqaaOGaee4AaS2aaSbaaSqaaiabicdaWiabikdaYaqabaGccqGHRaWkcqqGRbWAdaWgaaWcbaGaeGimaaJaeGymaedabeaakiabbUgaRnaaBaaaleaacqaIWaamcqaIYaGmaeqaaOGaey4kaSIaee4AaS2aaSbaaSqaaiabicdaWiabigdaXaqabaGccqqGRbWAdaWgaaWcbaGaeGymaeJaeGOmaidabeaaaaGcdaWadaqaauaabeqaciaaaeaacqqGRbWAdaWgaaWcbaGaeGimaaJaeGOmaidabeaakiabgUcaRiabbUgaRnaaBaaaleaacqaIXaqmcqaIYaGmaeqaaaGcbaGaee4AaS2aaSbaaSqaaiabigdaXiabikdaYaqabaaakeaacqqGRbWAdaWgaaWcbaGaeGOmaiJaeGymaedabeaaaOqaaiabbUgaRnaaBaaaleaacqaIWaamcqaIXaqmaeqaaOGaey4kaSIaee4AaS2aaSbaaSqaaiabikdaYiabigdaXaqabaaaaaGccaGLBbGaayzxaaGaaCzcaiaaxMaadaqadaqaaiabisda0aGaayjkaiaawMcaaaaa@83B6@

and the percentage R (%) of particles that recycle back after leaving the intracellular pool:

R=k21k21+k01⋅k12k12+k02     (5)
 MathType@MTEF@5@5@+=feaafiart1ev1aaatCvAUfKttLearuWrP9MDH5MBPbIqV92AaeXatLxBI9gBaebbnrfifHhDYfgasaacH8akY=wiFfYdH8Gipec8Eeeu0xXdbba9frFj0=OqFfea0dXdd9vqai=hGuQ8kuc9pgc9s8qqaq=dirpe0xb9q8qiLsFr0=vr0=vr0dc8meaabaqaciaacaGaaeqabaqabeGadaaakeaacqqGsbGucqGH9aqpdaWcaaqaaiabbUgaRnaaBaaaleaacqaIYaGmcqaIXaqmaeqaaaGcbaGaee4AaS2aaSbaaSqaaiabikdaYiabigdaXaqabaGccqGHRaWkcqqGRbWAdaWgaaWcbaGaeGimaaJaeGymaedabeaaaaGccqGHflY1daWcaaqaaiabbUgaRnaaBaaaleaacqaIXaqmcqaIYaGmaeqaaaGcbaGaee4AaS2aaSbaaSqaaiabigdaXiabikdaYaqabaGccqGHRaWkcqqGRbWAdaWgaaWcbaGaeGimaaJaeGOmaidabeaaaaGccaWLjaGaaCzcamaabmaabaGaeGynaudacaGLOaGaayzkaaaaaa@4B88@

Upper and lower bound were calculated for M_tot_, MRT_1 _and MRT_2_[22], while unique values were calculated for R.

### Model identifiability

Parameters k_21_, k_12_, k_01_, k_02_, and M_1 _of the model (figure 1) were fitted on disaturated-phosphatidylcholine-palmitate tracer to tracee ratio using SAAMII [24]. Weights were chosen optimally, i.e. equal to the inverse of the measurement errors. They were assumed to be Gaussian, independent and zero mean with a constant coefficient of variation, which was estimated a posteriori.

Masses of palmitate residues were multiplied by 1.3025 to obtain disaturated-posphatidycholine masses. Rate of changes (k), fluxes (F) and synthesis (P) were multiplied by 24 to obtain the respective values per day.

### Statistical analysis

Results are presented as mean ± SEM. Data in Table 1 are presented as mean ± SD. Differences were analysed using the Mann-Whitney test with a 2-tailed probability of <0.05 (SPSS 10.0, Windows 2000). Parameters, resolved as upper and lower bounds, were considered different when the interval of admissible values in ARDS was significantly different from the interval of admissible values in controls.

**Table 1:** Clinical characteristics of patients with ARDS and control subjects

	ARDS N = 12	CONTROLS N = 7	p
Body Weight (kg)	74 ± 16	58 ± 12	0.05
Age (years)	60 ± 16	50 ± 23	0.37
Mechanical Ventilation (days)	23 ± 16	81 ± 129	0.21
Mechanical Ventilation at the start of the study (days)	2.6 ± 2	69 ± 132	0.23
Male/Female (number)	8/4	3/4	0.324
Survival (alive/total number)	4/12	7/7	0.006
Mean FiO_2 _(percentage)	60 ± 16	24 ± 14	<0.001
Mean PEEP (cm H_2_O)	7.7 ± 1.8	1.3 ± 0.2	<0.001
Mean AaDO_2 _^§^	283 ± 129	52 ± 38	<0.001
Mean PaO_2_/FiO_2_*	162 ± 50	382 ± 79	<0.001

^§ ^AaDO_2 _= Mean Alveolar-arterial oxygen gradient during the study

* PaO_2_/FiO_2 _= PaO_2_/FiO_2 _ratio during the study period

Data is presented as mean ± SD

## Results

### Clinical characteristics

We studied 12 ARDS patients and 7 controls. No ARDS patient was treated with exogenous surfactant. Eight ARDS patients (67%) died before hospital discharge, 5 for multi-organ failure and 3 for the underlying disease. Patients died within 4 to 18 days of study completion and during the study respiratory and gas exchange parameters were stable. No death occurred in the control group. In the control group, five patients suffered from spinal muscular atrophy, two had polineuropathy and one had encephalopathy secondary to head injury. Clinical characteristics of the 12 ARDS and 7 controls are reported in Table 1. ARDS was induced by an indirect insult in all but one patient (patient 5, Table 2). Mean age was comparable in the two groups, mean weight was significantly lower in control groups (p = 0.05) and the male/female ratio was 8/4 in ARDS and 3/4 in controls (p = 0.26). Ventilator parameters were significantly different as expected from the study design. All ARDS patients were mechanically ventilated, whereas six controls were on intermittent ventilator support and one was breathing spontaneously via tracheostomy tube. Table 2 reports detailed clinical data for the 12 ARDS patients.

**Table 2:** Clinical characteristics of patients with ARDS

Patient	Sex	Weight (kg)	Age (years)	Intubation^‡ ^(days)	Survival (Y/N)	Main Diagnosis	PaO_2_/FiO_2_M/m* (%)	AaDO_2_M/mx^§ ^(mmHg)
Pt1	F	48	86	24/5	N	Gastric ulcer, MOSF^†^	221/171	140/159
Pt2	M	95	27	11/1	N	Polytrauma, MOSF^†^	136/82	423/575
Pt3	F	57	47	6/0	N	Rectal cancer, MOSF^†^	145/111	235/279
Pt4	M	88	69	33/3	N	Sepsis post pancreatectomy	132/70	382/482
Pt5	M	90	53	49/6	Y	Politrauma, lung contusions	153/63	399/608
Pt6	M	69	59	6/3	N	Gastrectomy, MOSF^†^.	82/62.	555/590
Pt7	M	88	62	15/1	Y	Sepsis	194/58	177/605
Pt8	F	52	46	47/3	Y	Cyrrosis, liver transplant	146/92	276/396
Pt9	M	78	71	42/5	Y	Candida Pneumonia	268/187	158/260
Pt10	M	70	61	11/4	N	Gastric Cancer	156/87	214/414
Pt11	F	60	69	18/0	N	Pancreatic Cancer	118/70	267/333
Pt12	M	88	74	13/5	N	Pancreatitis	195/129	173/227

‡ Intubation = number of days of intubation/days of intubation at the start of the study

† MOSF = Multi Organ System Failure

* PaO_2_/FiO_2 _M/m = PaO_2_/FiO_2 _ratio Mean/minimum during the study period

§AaDO_2_M/mx = Alveolar-arterial oxygen gradient Mean/maximum during the study period

### Kinetic calculations

**Figure 2:**
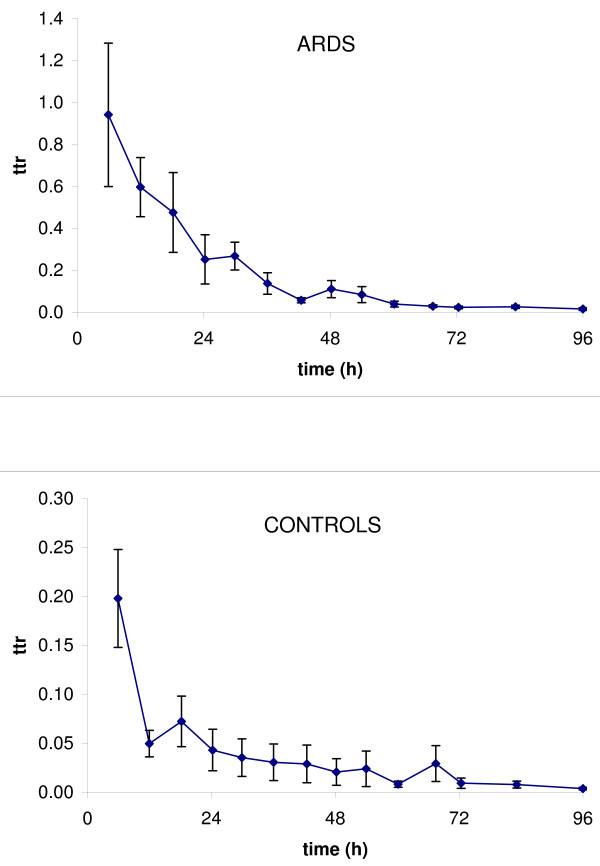
**Tracer to tracee ratio plot**. Tracer to tracee ratio (ttr) in disaturated-phosphatidylcholine and palmitate isolated from tracheal aspirates in ARDS (upper) and controls (lower). Values are mean ± SEM. n = 7 for control subjects and 12 for patients with ARDS.

The average time courses of disaturated-phosphatidylcholine-palmitate tracer to tracee ratio in controls and ARDS are shown in figure 2. Although similar tracer doses were used in ARDS and controls, the tracer to tracee ratio of ARDS was markedly higher than in controls. In both cases, the tracer to tracee ratio declined to negligible values at 96 h. Therefore we used data up to 96 h.

**Figure 3:**
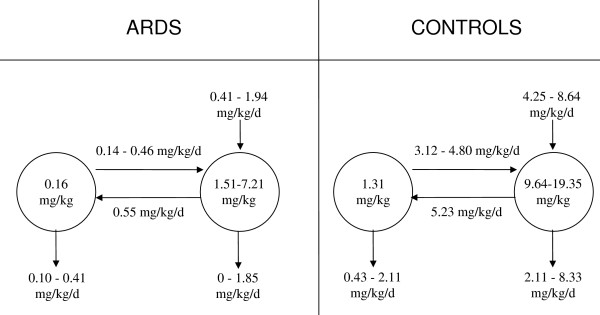
**Main kinetic results**. Disaturated-phosphatidylcholine-palmitate kinetics in ARDS (left) and controls (right). Unique values are estimated only for M_1 _and F_12_. Other parameters are presented as ranges, limited by average upper and lower bounds.

**Figure 4:**
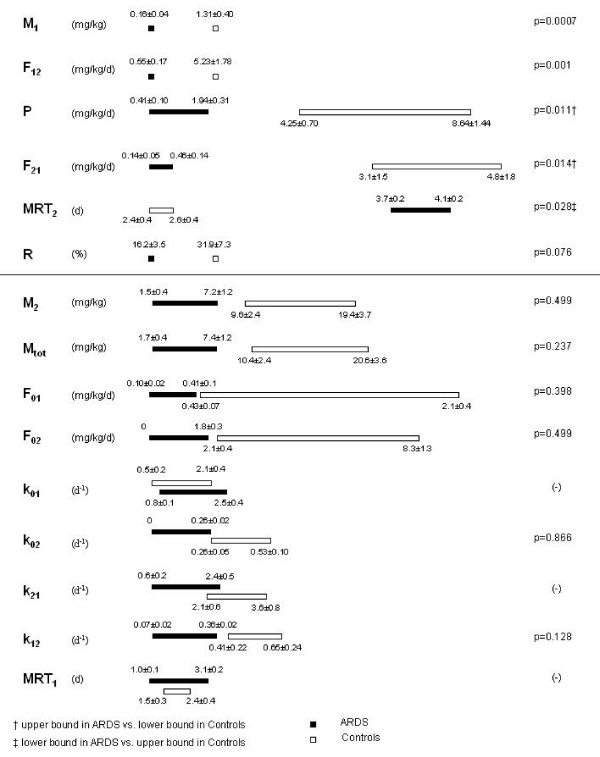
**Detailed kinetic results**. Estimated and derived kinetic parameters of ARDS patients (black boxes) and controls (white boxes). Values are expressed as mean ± SEM. Symbols as in figure [1]. Stars (*) represent unique values in ARDS that were significantly lower (p < 0.05) than the respective values in controls. Crosses (†) indicate intervals of admissible values in ARDS significantly lower than in controls (upper bound in ARDS significantly lower than lower bound in controls). Double crosses (‡) indicate intervals of admissible values in ARDS significantly higher than in controls (lower bound in ARDS significantly higher than upper bound in controls).

Individual curves of the tracer to tracee ratio were fitted to the model presented in figure 1. All parameters were estimated with acceptable precision, on average less than 50%. Kinetic parameters are summarized in figure 3 and depicted in greater detail in figure 4. Three of them (M_1_, F_12 _and R) were uniquely identified, the others are presented as ranges of values included between two extremes, the upper and lower bounds.

In controls, the alveolar pool of disaturated-phosphatidylcholine was 1.31 ± 0.40 mg/kg, far smaller than the tissue pool, which, depending on assumptions about degradation of disaturated-phosphatidylcholine by alveolar macrophages, ranged from 9.64 ± 2.43 to 19.35 ± 3.74 mg/kg. *De novo *synthesis (P) of disaturated-phosphatidylcholine ranged from 4.25 ± 0.7 to 8.64 ± 1.44 mg/kg/day. The flux from alveoli to tissue (F_21_) ranged from 3.12 ± 1.49 to 4.80 ± 1.78 mg/kg/day. The flux from tissue to alveoli (F_12_) was 5.23 ± 1.78 mg/kg/day and recycling (R) was 31.9 ± 7.3%. According to the model, labelled disaturated-phosphatidylcholine is expected to accumulate into the lung parenchyma of control subjects, reaching a maximum concentration between 12 and 24 hours after instillation. Afterwards, tissue isotopic enrichment is expected to decrease, so that 96 hours after the start of the study around 20% of the tracer remains associated with lung tissue (data not shown).

In patients with ARDS, the alveolar pool of disaturated-phosphatidylcholine (M_1_) was smaller than in controls: 0.16 ± 0.04 vs 1.31 ± 0.40 mg/kg (p < 0.05). Fluxes between tissue and alveoli (F_12 _and F_21_) and *de novo *synthesis (P) of disaturated-phosphatidylcholine were also smaller than in controls. Fractional rates of transfer between tissue and airways (k_21 _and k_12_) and alveolar mean resident time (MRT_1_) were not different from controls. In ARDS, the tissue mean resident time of disaturated-phosphatidylcholine was significantly longer than in controls (figure 3 and 4). Recycling tended to be smaller in patients with ARDS, but the difference was not significant: 16.2% ± 3.5 in ARDS and 31.9% ± 7.3 in controls (p = 0.08, figure 4). Differences between ARDS and control patients appear to be robust, since, with the exception of the synthesis rate P, all differences remained significant even assuming that in controls 5–100% of disaturated-phosphatidycholine can be degraded in the alveolar spaces.

The model predicts that in ARDS instilled disaturated-phosphatidylcholine associates rapidly with lung tissue, reaching a maximum after 12–24 hours, and then decreases gradually, so that after 96 hours 10–30% of the dose remains tissue-associated (not shown).

## Discussion

Pulmonary surfactant is essential for normal lung function, and it is well established that surfactant impairment contributes to respiratory failure in ARDS [4,5,25-27]. These observations prompted the use of exogenous surfactant in ARDS, to replenish a deficient state and reverse surfactant inactivation [28-30]. However, large randomized clinical trials have given puzzling results [28-30] suggesting that other processes, besides surfactant dysfunction, may contribute to lung damage in ARDS or at least indicating that exogenous surfactant is either rapidly inactivated or is preferentially distributed to normal lung sections.

Most of our knowledge on surfactant kinetics in acute lung injury derives from animal studies done with radioactive isotopes [7]. In this study we analysed the turnover of surfactant disaturated-phosphatidylcholine in control subjects and in patients with ARDS using stable isotopes. The technique used has been validated in pre-term baboons with bronchopulmonary dysplasia. In that experiment we found that the estimate of the alveolar and tissue pools of disaturated phosphatidylcholine obtained from the dilution of stable isotopes in tracheal aspirates compared well with direct measurements done at autopsy. [31]. The technique has been also applied to human infants with neonatal respiratory distress syndrome due to prematurity, lung malformations and infections [18,32-36]. However there are aspects of the present work, both conceptual and technical, that warrant special comment.

### Basic assumptions

The design of the study assumes that the tracer was administered as a pulse, that there was good mixing between tracer and endogenous surfactant, that the administered material did not perturb endogenous surfactant, that tracheal aspirates were representative of events happening in the most peripheral airways and that patients were at steady state.

While in neonatal respiratory disorders the lung parenchyma is relatively homogeneous, this is certainly not the case in patients with ARDS, where areas of atelectasis and over-distension coexist and the tracer might distribute preferentially to aerated sections of the lungs [3]. In this study, to optimize distribution, we mixed the tracer with a surfactant extract used as a spreading agent. We could not document directly in our patients that the instilled material distributed uniformly throughout the aerated airways, but we relied on the following findings all indicating that the instilled material mixed well with resident surfactant: a) animals who receive surfactant through the airways with the technique we used, display a rather homogeneous distribution through the airways, [37-39]; b) our estimate of the alveolar pool of disaturated-phosphatidylcholine in control patients agrees very nicely with data obtained by Rebello et al on bronchoalveolar lavage fluid of human cadaver lungs [40]; c) in preterm baboons we found that the disaturated-phosphatidylcholine pools calculated from the dilution of tracers administered through the trachea compare well with direct measurements done at autopsy [31]; d) in the same experiment we found the disaturated-phosphatidylcholine tracer enrichments in tracheal aspirates were remarkably similar to the enrichments measured in the bronchoalveolar lavage fluid (data not shown).

The dose of disaturated-phosphatidycholine administered to control subjects (20 ± 2 mg) represented 1.1–2.1% of the estimated lung pool [5], an amount unlikely to perturb endogenous surfactant. In patients with ARDS, the dose (20 ± 2 mg) represented 5.0–13.1% of the estimated lung pool, an amount also unlikely to induce a pharmacologic effect, considering that the doses of surfactant used for the treatment of ARDS are at least two orders of magnitude greater [29,30]. Since the dose of surfactant administered was small and clinical conditions remained stable during the study, we assume that the system was at steady state, thus allowing the use of a linear time invariant compartmental model to describe disaturated-phosphatidylcholine kinetics.

Data were analysed according to the two compartment model reported in figure 1. This model is physiologically plausible, but too complex to be uniquely resolvable from the available data, since only the mass in the alveolar compartment (M_1_), the flux from the lung tissue back to the alveolar space (F_12_) and recycling (R) can be uniquely solved. Only a far more complex experiment, with tracer administered also in the lung tissue compartment, could permit to uniquely identify all kinetic parameters. Since this experiment could not be done, we used existing knowledge on the contribution of alveolar macrophages to surfactant degradation to derive bounds for parameters that could not be uniquely identified. Thus, on the basis of animal experiments done by Gurel and Rider [10,11], we assumed that alveolar macrophages could normally degrade between 5 and 50% of surfactant disaturated-phosphatidylcholine, the remaining being degraded by the lung parenchyma. It should be noted however, that 50% degradation by alveolar macrophages probably represents a maximum, since this figure was derived on the assumption that alveolar macrophages do not re-enter lung parenchyma after the uptake of surfactant in the alveoli [10]. In ARDS, we assumed that 5–100% of surfactant disaturated-phosphatidylcoline could be degraded in the airways, due to the degradative activity of inflammatory cells or bacteria. By incorporating these assumptions into the tracer-tracee model, upper and lower bounds were derived for all non identifiable kinetic parameters, following a strategy formalized in [23] and applied to study thyroid hormones [41,42] and glucose [43] kinetics.

### Surfactant kinetic parameters in controls

Our estimate of the alveolar and tissue pools of disaturated-phosphatidylcholine in controls agree quite well with measurements taken by Rebello et al. during autopsies of adults without lung disease [40]. In fact, according to Rebello et al. the alveolar and tissue pools contain respectively 1.9 μmol/kg and 28.4 μmol/kg of disaturated-phosphatidylcholine. We found that in controls the alveolar pool of disaturated-phosphatidylcholine was 2.3 μmol/kg, while the tissue pool ranged between 17.1 and 34.3 μmol/kg. It is also of note that our results compare favorably with those of Martini et al. who studied surfactant turnover in adult pigs using stable isotopes [44]. These authors reported that mean phosphatidylcholine synthesis was 4.7 mg/kg/day, while our estimate ranged between 4.3 and 8.6 mg/kg/day. Furthermore they reported that the phosphatidylcholine tissue pool was 10 times higher than the alveolar pool [44], in good agreement with our finding that in control subjects the tissue pool was 7.6–14.8 times greater the alveolar pool. Overall, these results support our approach and also indicate that tracheal aspirates can be as useful as bronchoalveolar lavage fluid for the study of surfactant turnover.

Using morphometric methods Young et al. estimated that the alveolar pool of disaturated-phosphatidylcholine is comparable to the lamellar body pool [45]. Thus it is likely that the tissue pool of disaturated-phosphatidylcholine measured with the present technique includes both intracellular surfactant and non-surfactant membranes that, with time, incorporate a fraction of administered disaturated-phosphatidylcholine.

### Surfactant in ARDS

In patients with ARDS alveolar pool, fluxes between tissue and alveoli and *de novo *synthesis of disaturated-phosphatidylcholine were all smaller than in controls, while the mean residence time in lung tissue was greater than in controls. These differences appear to be robust, since, with the exception of de novo synthesis, they persist even assuming that in controls alveolar macrophages degrade between 5% and 100% of surfactant disaturated-phosphatidylcholine. Thus most of our conclusions remain valid independent of any assumption regarding the site of degradation of surfactant.

The present results agree with the view that, in ARDS, only a fraction of the lung is accessible to exogenous surfactant. In fact, the decrease of the alveolar pool of disaturated-phosphatidylcholine, the decrease of fluxes between tissue and alveoli and the decrease in the rate of synthesis can all be interpreted assuming that instilled surfactant reached only aerated lung sections. However, our data do not support the notion that these residual lung sections were normal, since the mean resident time of disaturated-phosphatidylcholine in lung parenchyma (MRT_2_) was greater while the rate of recycling tended to be lower than in controls. The greater mean residence time of disaturated-phosphatidylcholine in lung tissue could be due to a number of factors, namely to a decreased ability to degrade surfactant components, to an increased reacylation of lysophosphatidylcholine (favored by the increased availability of palmitate residues generated by phospholipase A_2_, released by inflammatory cells), to a proliferation of type II cells, to the distribution of tracer to lung structures not pertaining to the surfactant system (i.e. infiltrating inflammatory cells), or to a combination of these phenomena [46]. The distribution of tracer to lung structures not pertaining to the surfactant system could explain the tendency towards a less efficient recycling of DSPC observed in patients with ARDS (figure 4).

## Conclusion

Surfactant pool size is greatly diminished in ARDS compared to control, and surfactant kinetics is altered in ARDS resulting from a significantly reduced production rate and a significantly longer retention time in the 2nd (tissue) compartment.

The fact that the alveolar pool of disaturated-phosphatidylcholine can be estimated unambiguously is an important result of this work. In future studies this approach could be used to relate changes in surfactant turnover with time course and severity of ARDS or to evaluate the effect of different treatments (ventilation modes, inhaled or intravenous therapies) on surfactant metabolism.

## Abbreviations

ARDS = acute respiratory distress syndrome

k_21 _and k_12 _= disaturated-phosphatidylcholine inter-conversion rate parameters,

k_01 _and k_02 _= disaturated-phosphatidylcholine irreversible losses,

u = labeled disaturated-phosphatidylcholine-palmitate injection into the accessible compartment.

M_1 _= the alveolar pool of disaturated-phosphatidylcholine

M_2 _= the tissue pool of disaturated-phosphatidylcholine

M_tot_= total disaturated-phosphatidylcholine pool

F_21_, F_12_, F_01_, F_02 _= disaturated-phosphatidylcholine inter-conversion and irreversible loss fluxes in compartment 1 (alveoli) and 2 (tissue)

P = *De novo *synthesis of disaturated-phosphatidylcholine

MRT_1 _and MRT_2 _= mean residence time of disaturated-phosphatidylcholine in compartment 1 (alveoli) and 2 (tissue)

## Competing interests

The author(s) declare that they have no competing interests.

## Authors' contributions

PEC participated to the design and coordination of the study and drafted the manuscript. GMT, MC, CC performed the data modeling and analysis. CO and AV were responsible of the clinical conduction of the study. AG performed the mass spectrometry analysis. BA and VPC participated in the study design and helped to draft the manuscript.
